# Effects of Chinese wolfberry and Astragalus extract on the antioxidant capacity of Tibetan pig liver

**DOI:** 10.1371/journal.pone.0245749

**Published:** 2021-01-27

**Authors:** Zhuang Hao, Zhen Li, Jinjin Huo, Jiandong Li, Fenghua Liu, Peng Yin

**Affiliations:** 1 College of Animal Science and Technology, Beijing University of Agriculture, Beijing, China; 2 Institute of Microbiology Chinese Academy of Sciences, Beijing, China; University of Naples Federico II, ITALY

## Abstract

The objective of this study is to determine the effect of Chinese wolfberry (Lycium barbarum) and Astragalus (Astragalus membranaceus) extract (WAE) on the antioxidant capacity of Tibetan pig liver, and discussed the regulatory effect of WAE on the liver antioxidant mechanism. Twelve healthy 120-day-old Tibetan black pigs (35±2 kg) were divided randomly into two groups. The WAE group was fed a basal diet supplemented with 1% WAE for 90 days. The control group was fed the same diet, but without the WAE. We found that liver superoxide dismutase 1 (SOD1) activity (P<0.05), total antioxidative capacity (T-AOC) (P<0.05), and catalase (CAT) activity (P<0.01) significantly increased in the WAE group compared with the control group; malondialdehyde (MDA) content decreased, but this was not significant (P >0.05). Transcriptome sequencing analysis detected 106 differentially expressed genes (DEGs) related to oxidative stress. GO enrichment analysis showed these DEGs were involved in the positive regulation of reactive oxygen metabolism and biosynthesis, process regulation, and regulation of the oxidative stress response. KEGG Pathway enrichment analysis showed they were enriched in the PI3K-Akt, AMPK, Rap1, and peroxisome signaling pathways. The expression levels of key peroxisome biosynthesis genes (e.g., PEX3 and PEX11B) and key antioxidant genes (e.g., CAT and SOD1) were significantly higher in the WAE group than in the control group. The PRDX1 and PRDX5 content also was significantly higher in the WAE group. This study showed that the WAE regulated the antioxidant and anti-stress ability of Tibetan pig liver through a “peroxisome antioxidant-oxidant stress” signaling pathway.

## Introduction

In modern pig production, stress is an important factor that affects performance and other traits, and oxidative stress is a common type of stress. Oxidative stress disrupts the body’s redox balance and increases the production rate of reactive oxygen species (ROS). This results in the accumulation of large numbers of oxygen free radicals that can cause cell damage and apoptosis, and is closely related to many diseases [1, 2]. With the rapid development of intensive pig breeding methods, factors such as high temperature, harmful gases, high-fat and high-protein diets, and changes in feeding methods can all cause oxidative stress, thereby destroying the body’s antioxidant defense system and causing fattening pigs to decline in health and production performance, resulting in serious economic losses. The main function of the liver is metabolism and synthesis. Liver cells contain many thousands of mitochondria and are the main site of redox reactions. Liver produces large amounts of ROS and also is the main organ attacked by ROS [3]. Liver cells also contain many peroxisomes, which play important roles in the production of ROS and the metabolic pathways closely related to oxidative stress [4, 5].

Antioxidants have strong free radical scavenging activity and some clinical antioxidation benefits in livestock and poultry. Synthetic antioxidants have been linked to harmful effects, such as liver damage [6]; therefore, bioactive ingredients and extracts from natural sources such as medicinal plants are of great interest as safe and effective antioxidants [7, 8]. Chinese wolfberry (Lycium barbarum) and Astragalus (Astragalus membranaceus) have been recorded in Chinese classical literature, and both are considered to have high medicinal value. Wolfberry contains a variety of health ingredients, including carotenoids, betaine, vitamins, and polysaccharides, which are the main ingredient [9]. These ingredients have been reported to have antioxidant, anti-inflammatory, anti-tumor, anti-stress, anti-diabetic, liver protection, and immune regulation properties [10–14]. Lycium barbarum polysaccharides (LBP) have been found to reverse the oxidative stress induced by weaning, improve the intestinal health of pigs and piglets, promote the growth of beneficial intestinal bacteria, inhibit the growth of *Escherichia coli*, enhance the body’s immune function and antioxidant capacity, and improve growth performance [15]. Astragalus is a legume and dried Astragalus roots contain alkaloids, glycosides, flavonoids, amino acids, saponins, trace minerals, and polysaccharides, which are the main active ingredient. Astragalus polysaccharides have immunomodulatory and anti-virus, liver protection, hematopoiesis, neuroprotection, and anti-inflammatory activities [16–18]. It has been shown that Astragalus polysaccharides reduce the production of ROS in muscle cells and endothelial cells by maintaining the stability of the mitochondrial respiratory chain [19, 20]. These findings show that wolfberry and Astragalus both have antioxidant effects, and may be used as antioxidants in pig breeding. The ethanol extract of Astragalus and licorice was shown to have strong free radical scavenging ability, and the ethyl acetate extract of Astragalus and licorice had an obvious synergistic antioxidant effect and better cytoprotective effect than either of the single herbs [21, 22]. However, there are very few reports about the antioxidant effects of wolfberry and Astragalus extracts in Tibetan pigs.

Transcriptome sequencing (RNA-seq) is a new cutting-edge technology which enables quantitative analysis of a whole transcriptome. This technique has been used several times in exploring porcine transcriptomes from different tissues [23–27]. RNA-seq has been used to study oxidative stress and other related issues. We applied RNA-seq to study the effect of a wolfberry and Astragalus extract on gene expression in liver tissues of Tibetan pigs. Differentially expressed genes related to oxidative stress were identified and functionally annotated with KEGG pathways (https://www.kegg.jp/kegg/pathway.html) and GO terms to determine their significance. RNA-seq was used to discuss in depth the regulation of WAE on liver antioxidant mechanisms. We found that a “peroxisome antioxidant-oxidant stress” signaling pathway regulated the antioxidation and anti-stress ability of Tibetan pig liver.

## Materials and methods

### Animals and diet

All protocols involving animals were conducted in accordance with the standards approved by Beijing Administration Office of Laboratory Animal (approval number: SYXK 2015–0004).

Twelve healthy 120-day-old Tibetan black pigs weighing 35±2 kg were divided randomly into two groups, each with six pigs. The control group was fed a basic diet as shown in S1 Table. The treated group was fed the basic diet supplemented with 1% wolfberry and Astragalus extract (WAE) for 90 days. Chinese wolfberry and Astragalus were purchased from the An Guo medicine market in China (Anguo City, Baoding City, Hebei Province). The wolfberry and astragalus were mixed in a ratio of 1:1, 1kg in total, add ten times the amount of water to decort for six hours, dry, crush and mix, then extract with 95% ethanol at a ratio of 1:8 for six hours, then filter. the filtrates were concentrated on a rotary evaporator (RE-52AA, Yarong Biochemical Analysis Co., Ltd., Shanghai, China) (50°C) with a vacuum pump and freeze-dry the WAE. During the experimental period, all the groups were fed in a pigsty under the same feeding conditions, and were free to eat and drink. Before the experiment, the trough and the water tank were fully disinfected, and the pigs were immunized and dewormed according to the normal procedures.

### Liver sample collection

At the end of the experiment, all pigs were transported to the slaughter house. The right liver samples of the three pigs in each group were collected. One part was stored in the refrigerator at -20 °C for the determination of antioxidant indicators; the other part was placed in a cryotube. It was quickly put into liquid nitrogen, then transported back to the laboratory and stored in a -80°C refrigerator for transcriptome sequencing. The remaining three pig liver tissue pieces in each group were placed in cryopreservation tubes, quickly frozen in liquid nitrogen, and transported back to the laboratory to be stored in a -80°C refrigerator for qRT-PCR to DEGs.

### Determination of antioxidant indexes and antioxidant enzymes

Malondialdehyde (MDA), total antioxidative capacity (T-AOC), superoxide dismutase 1 (SOD1), and catalase (CAT) activities in the liver samples were determined using commercial kits (Jiancheng Institute of Biological Technology, Nanjing, Jiangsu, China) according to the manufacturer’s instructions. Peroxidase 1 (PRDX1) and peroxidase 5 (PRDX5) content in the samples were determined using Enzyme Linked Immunosorbent Assay (ELISA) kits (Wuhan Genemei Biotechnology Co, Ltd, Wuhan, Hubei, China).

### RNA extraction, library construction, and sequencing

The library construction and sequencing were performed at the Beijing Compson Biotechnology Company. RNA was extracted from the liver samples using TRIzol reagent (Invitrogen, United States USA). and quality was checked using Nano Drop (Thermo scientific, United States USA). 3 μg of RNA per sample was used as input material for the RNA sample preparations. All six RNA samples had RNA integrity number (RIN) values above eight. The libraries were sequenced using Illumina High-seq 2000 technology. Sequencing libraries were generated using an Illumina TruSeq RNA Sample Preparation Kit (Illumina, San Diego, USA) following manufacturer’s instructions. Briefly, mRNA was purified from total RNA using poly-T oligo attached magnetic beads.

Fragmentation was performed using divalent cations under an elevated temperature in Illumina proprietary fragmentation buffer. First strand cDNA was synthesized using random oligonucleotides and Super Script II. Second strand cDNA synthesis was performed using DNA polymerase I and RNase H. Overhangs were converted into blunt ends via exonuclease/polymerase activities and enzymes removed. After adenylation of 3′ ends of DNA fragments, Illumina PE adapter oligonucleotides were ligated to prepare for hybridization. The library fragments were purified using an AM Pure XP system (Beckman Coulter, Beverly, USA). DNA fragments with ligated adaptor molecules on both ends were selectively enriched using Illumina PCR Primer Cocktail in a ten cycle PCR reaction. Products were purified (AM Pure XP system) and quantified using the Agilent high sensitivity DNA assay on an Agilent Bioanalyzer 2100 system (Santa Clara, CA, USA). Clustering of indexcoded samples was performed on a cBot Cluster Generation System using TruSeq PE Cluster Kit v3-cBot-HS (Illumina) according to manufacturer’s instructions. After cluster generation, the library preparations were sequenced on an Illumina Hiseq TM 2500 platform, and 100 bp paired-end reads generated.

### Mapping of the reads and transcripts assembly

The porcine reference genome and annotated transcript set were downloaded from *Scrofa* 11.1 of Ensembl (http://www.ensembl.org/). The index of the reference genome was built using Bowtie v0.12.8 [28], and paired-end clean reads were aligned with the reference genome using TopHat v1.4.0 [29]. The Cufflinks v1.3.0 Reference Annotation Based Transcript assembly method was used to construct and identify both known and novel transcripts from the TopHat alignment results [30].

#### Quantification and clustering of differentially expressed genes

The reads amounts that mapped to each gene were counted by HTSeq v0.6.1. And then the number of Fragments Per Kilobase of transcript sequence per Millions base pairs sequenced (FPKM) was calculated [31]. Differential expression analysis of two groups were performed using the DESeq 2 (1.20.0). The P values were adjusted using the Benjamini and Hochberg method [32]. Corrected P value (q value) of 0.05 and log2 (Fold change) of ≥1 were set as the threshold for significantly differential expression. In addition, the hierarchical method was used for clustering analysis of differentially expressed genes. The significance of the differentially expressed genes (DEGs) was determined by KEGG and gene ontology (GO) enrichment analyses, and by constructing a gene co-expression network based on the DEGs.

### Quantitative PCR (qPCR)

Total RNA was extracted from the liver tissue using a total RNA kit (Tiangen, Beijing, China). The qPCR analysis was carried out using the DNA Engine Mx3000P^®^ (Agilent, CA, USA) fluorescence detection system against a double-stranded DNA specific fluorescent dye (Stratagene, CA, USA) according to optimized PCR protocols. ACTB was used as the normalization control. The PCR cycles were 95°C for 3 min, followed by 40 cycles of 95°C for 15 s, 60°C for 30 s, and 72°C for 60 s. The genes selected for validation by qPCR included peroxisome biogenesis factors PEX3, PEX11B, PEX16, and PEX19, peroxidases PRDX1 and PRDX5, catalase (CAT), superoxide dismutase 1 (SOD1), heat shock protein 90 family A member 1 (HSP90AA1), Parkinson’s syndrome related deglycase 7 (PARK7), HAO1, HADH, DECR1, ACADVL, SESN2, and MSRB1. The gene-specific oligonucleotide primers used for the qPCRs are listed in S2 Table.

### Data statistics and analysis

The RNA-seq data were analyzed statistically with DESeq 2 and the other data were analyzed by the one-way T-test in SPSS for Windows (version 20.0; SPSS Inc., Chicago, IL, USA). The results were expressed as means ± standard deviation (SD). Transcriptome data difference analysis screening criteria were biological replicates adjusted P-value <0.05 and no biological replicates |log2FC|>1. The enrichment analysis screening criterion was adjusted P-value <0.05.

## Results

### Enzyme activity in the liver tissue

**Fig 1:**
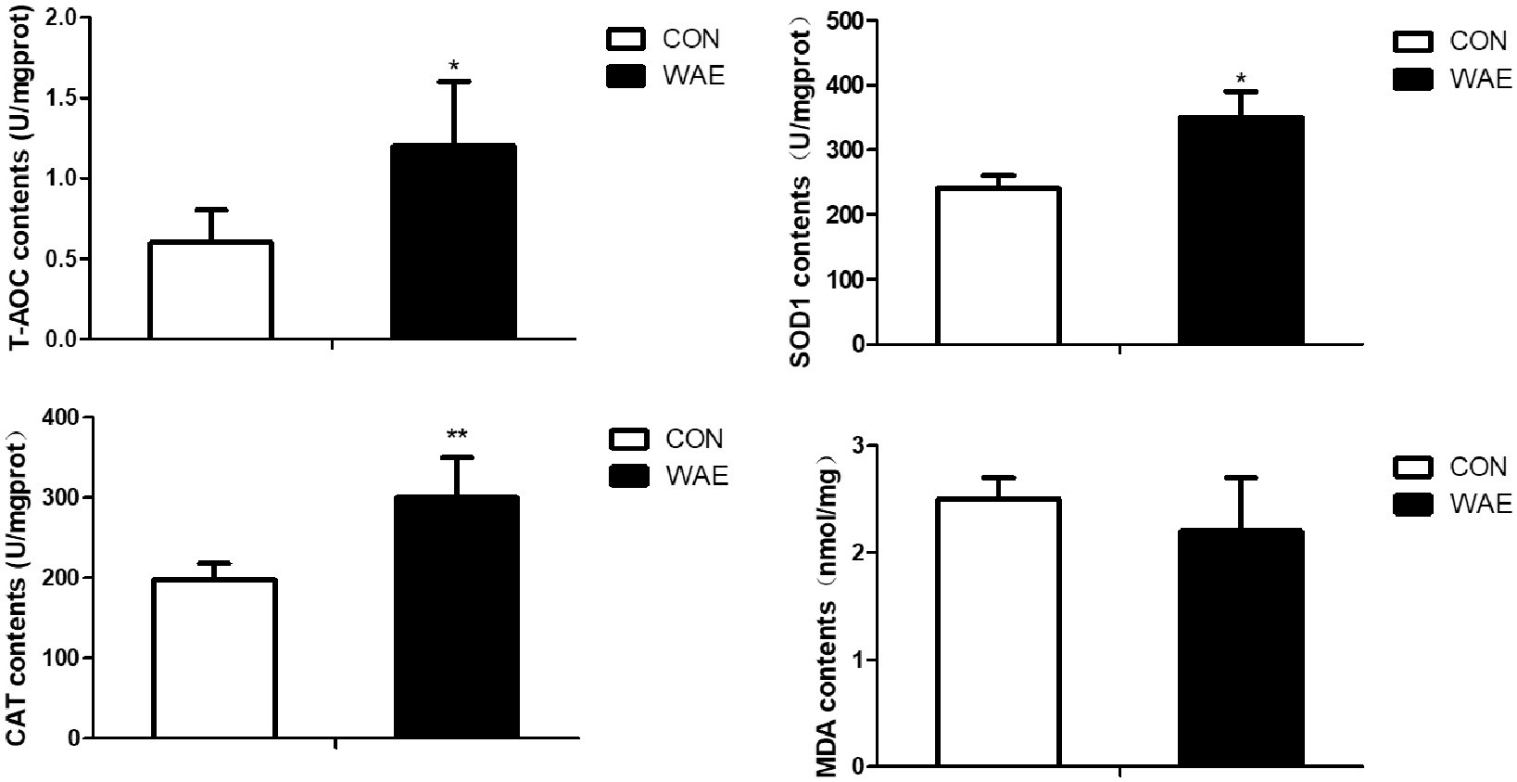
Effect of WAE on anti-oxidation of Tibetan pig liver. CON: basal diet. WAE: basal +1‰ WAE. T-AOC: total antioxidant capacity; SOD1: superoxide dismutase 1; CAT: catalase; MDA: malondialdehyde; *: (P<0.05) **: (P<0.01) ***: (P<0.001).

Dietary WAE supplementation led to significantly higher SOD1 and CAT activity and T-AOC (P <0.01), and lower MDA content (P >0.05) in the liver compared with in the control samples (Fig 1).

### Mapping reads to the transcriptome

We obtained 47.89 Gb of raw sequencing data and 46.14 Gb of clean data after filtering. The volume and quality statistics of the sequencing data for all the samples are given in Table 1.

**Table 1:** Statistical table of sequencing data quantity and quality.

Sample	Raw reads	Clean reads	Raw bases	Clean bases	Effective Rate	Error Rate	Q20,Q30	GC Content
C1	29266751	28766311	8.51	8.34	96.34	0.02	98.68	96.45
C2	30288811	29234721	9.09	8.77	96.52	0.02	98.79	96.36
C3	28241224	27222012	8.47	8.17	96.39	0.02	98.84	96.47
W1	26212528	25659283	7.86	7.7	97.89	0.02	98.62	96.06
W2	23999101	23117734	7.2	6.94	96.33	0.02	98.44	95.78
W3	23903259	22431466	7.17	6.73	93.84	0.03	97.66	94.04

Raw reads: number of raw reads sequenced; Clean reads: number of clean reads after filtering; Raw bases: the total number of bases in the original sequence data, that is, the number of raw reads multiplied by the length of the sequence, the unit is G; Clean bases: the total number of bases in the filtered date, the unit is G; Effective Rate: base sequencing efficiency; Error Rate: average base sequencing error rate; Q20,Q30: the percentage of bases with a Phred value greater than 20 or 30 to the total number of bases; GC Content: the percentage of bases with G/C in total bases.

### DEGs analysis

**Fig 2:**
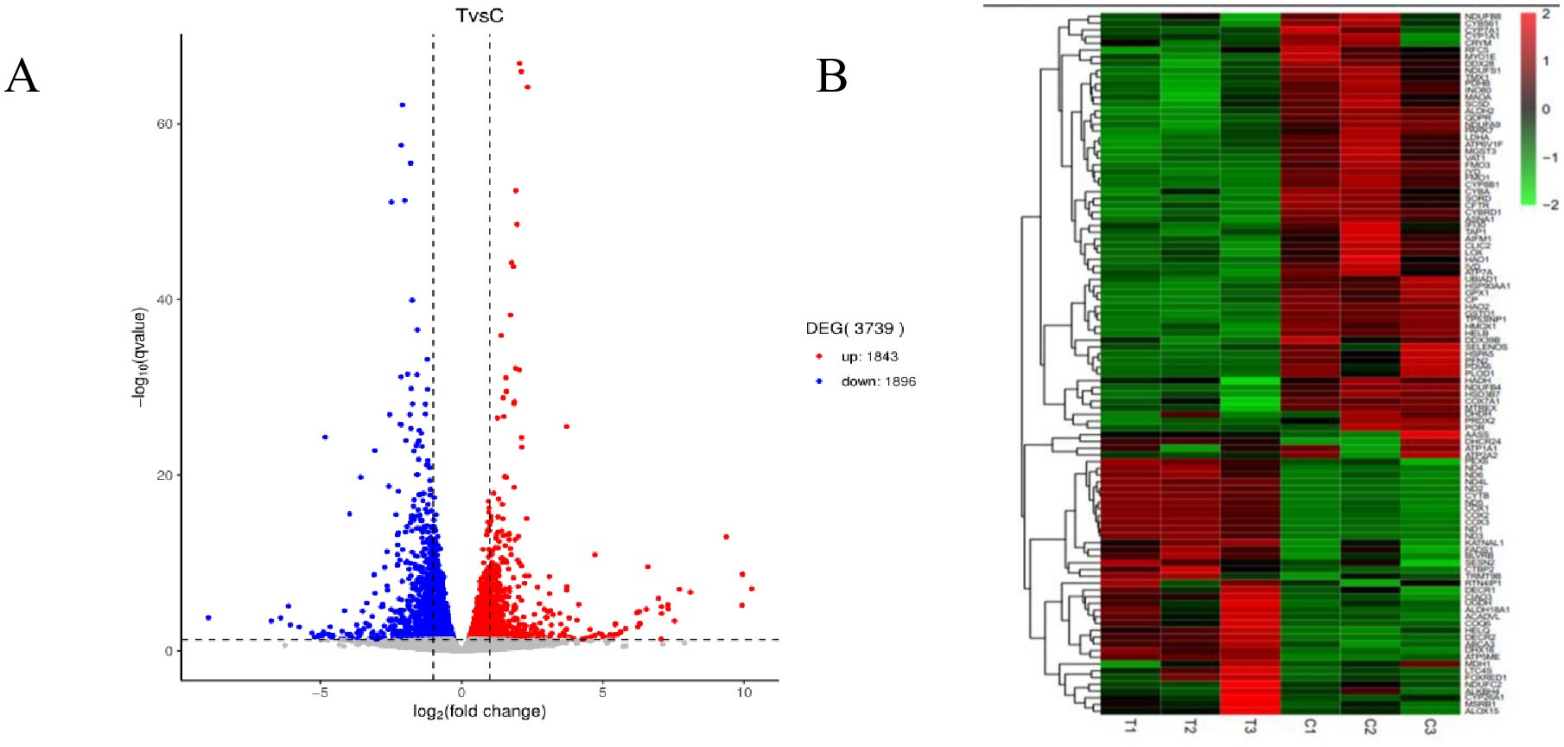
Analysis of differential genes in liver of WAE group and CON group. (A) Volcano map: Genes with significant differential expression were represented by red dots (up-regulated) and blue dots (down-regulated), and genes with no significant differentially expression were represented by gray dots; The abscissa represented the fold change of gene expression in different samples; the ordinate represented the statistical significance of the difference in gene expression. (B) The DEGs cluster map: red is significantly up-regulated gene, green was a significantly down-regulated gene, and regions with different colors represent different clustering grouping information.

**Fig 3:**
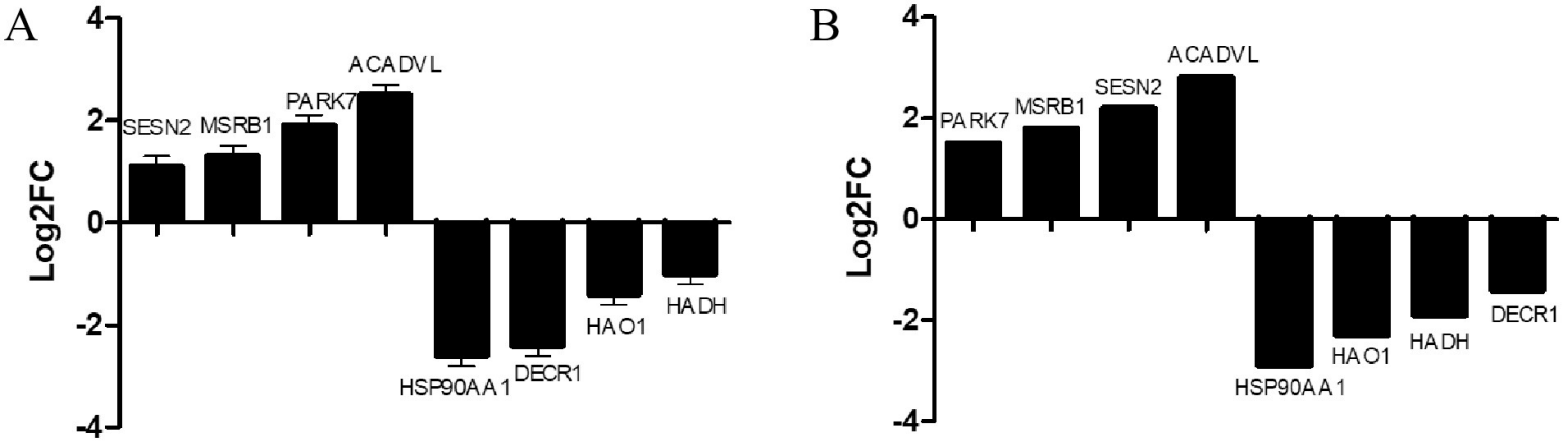
RNA-seq and qRT-PCR verification results of 8 DEGs in liver tissue. (A) qRT-PCR verification results of 8 DEGs in liver tissue. (B) RNA-seq results of 8 DEGs in liver tissue. Those on the X-axis are up-regulated genes, and those on the X-axis are down-regulated genes. Data are the means ± SD of three parallel experiments.

To determine the effect of the WAE on liver antioxidant genes in Tibetan black pigs, we used DESeq2 software to analyze the DEGs in the livers of the control and WAE groups. We detected 3739 DEGs (P <0.05 and |log2FC| ≥1) between the two groups; 1843 were up-regulated and 1896 were down-regulated as shown in the volcano plot in Fig 2A. Screening and functional annotation detected 106 DEGs related to oxidative stress; 42 were up-regulated and 64 were down-regulated. The gene expression sequence tags (EST) are shown in S3 Table. Cluster analysis of different groups of genes (Fig 2B) showed that similar genes had similar expression patterns, indicating that these DEGs may have similar functions or may be involved in the same pathway. The results showed that the samples in a group had good parallelism, and the difference between the groups was significant. To validate the RNA-seq results, we randomly selected eight DEGs (SESN2, MSRB1, PARK7, ACADVL, HSP90AA1, DECR1, HAO1, and HADH) and determined their expression by qPCR. The results showed that the qPCR expression trends were consistent with the RNA-seq results (Fig 3).

### GO and KEGG Pathway enrichment analysis of the DEGs

**Fig 4:**
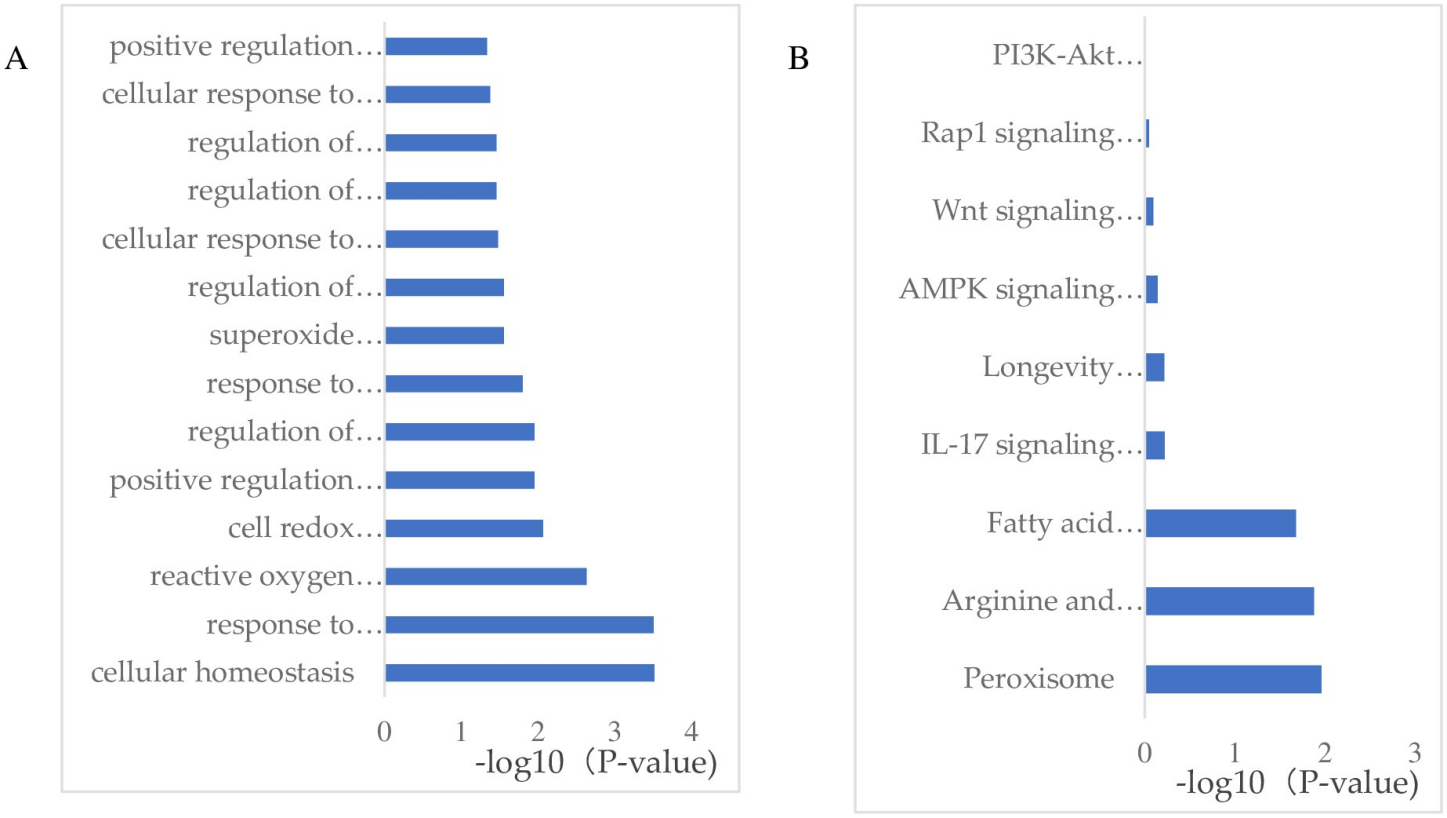
GO and KEGG Pathway enrichment analysis of the DEGs. (A) GO enrichment analysis, the Y coordinate in the fig is the GO item name, and the X coordinate is the -log10 (P-value). (B) KEGG enrichment analysis, the Y axis in the fig is the Pathway name, and the X coordinate is the -log10 (P-value).

To investigate the differences in liver biological processes between the WAE and control groups, we performed GO and KEGG Pathway enrichment analyses of the DEGs to determine the biological functions and signal pathways that they were associated with. The GO enrichment analysis showed that the DEGs were enrichment in positive regulation of reactive oxygen metabolism, regulation of reactive oxygen biosynthesis, regulation of oxidative stress, cells response to oxidative stress, regulation of oxidoreductase activity, peroxide metabolism, endoplasmic reticulum stress response, regulation of reactive oxygen species, positive regulation of oxidoreductase activity, oxidative stress response, cellular homeostasis, cellular redox homeostasis, and reactive oxygen species as shown in Fig 4A.

Generally, DEGs act together to perform their biological functions, so the most important biochemical metabolic and signal transduction pathways involving these genes can be determined through pathway enrichment analysis. We found that the DEGs were enriched in the PI3K-Akt, AMPK, Rap1, peroxisome, Wnt, and IL-17 signaling pathways, and fatty acid metabolism as shown in Fig 4B.

### Construction of a gene co-expression network based on the DEGs

**Fig 5:**
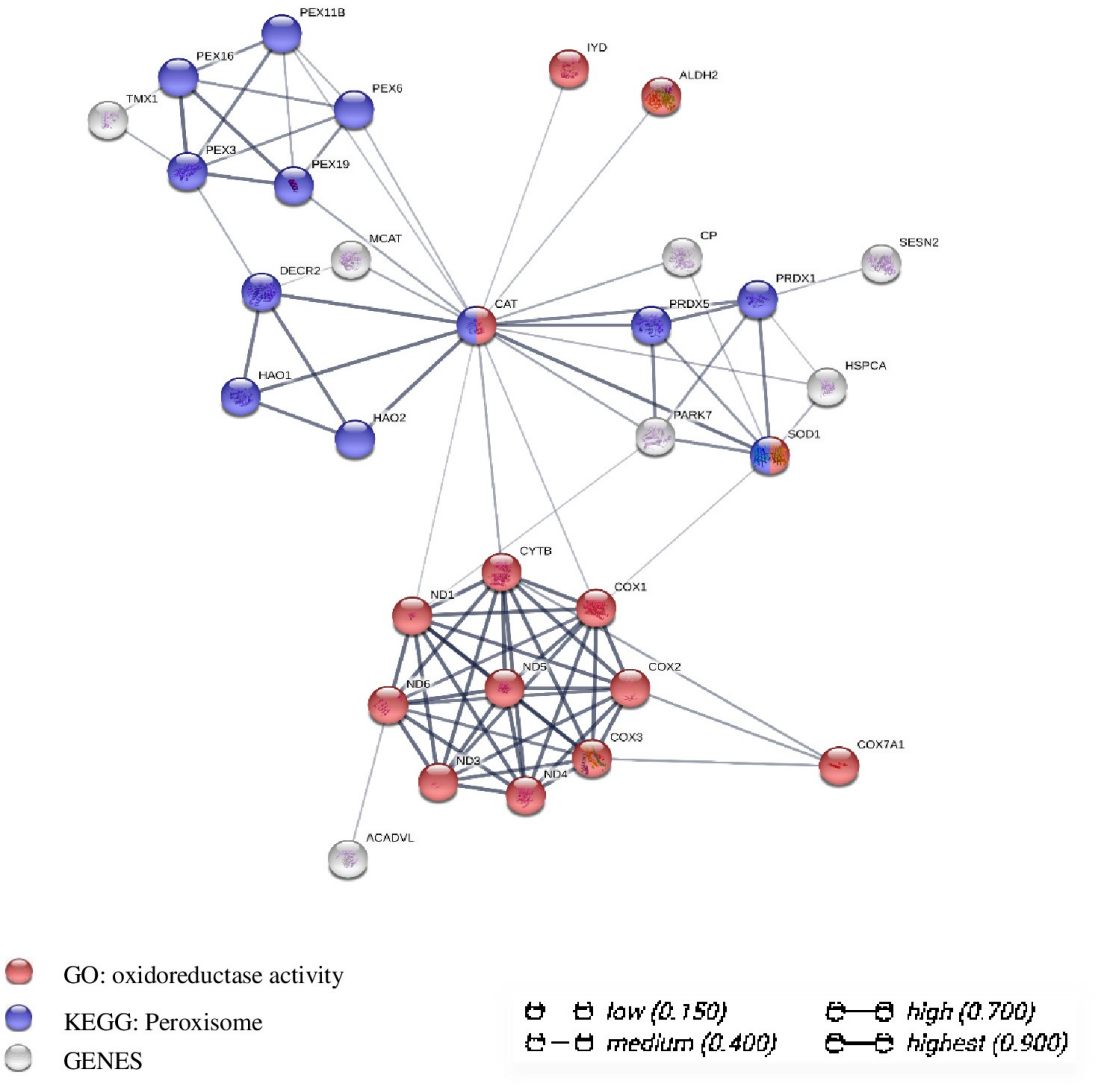
Analysis diagram of gene co-expression network interaction of DEGs between WAE group and control group. Each node represents all the proteins produced by a coding gene; the correlation of proteins is specific and meaningful, for example, proteins contribute to a common function.

To extract further relevant information from the transcriptome data, we constructed a gene co-expression network for the 106 DEGs. The network consisted of 23 nodes, 134 edges, and 5 significantly enriched pathways/biological processes related to oxidative stress. We identified PEX3, PEX11B, PEX16, CAT, SOD1, PRDX1, and PRDX5 as the hub genes and focused on the interaction of 31 key genes in the network, as shown in Fig 5. On the basis of these results, we speculate that the WAE affected key peroxisome biosynthesis genes such as PEX3, PEX11B, and PEX16 and key peroxisome antioxidant genes such as CAT, SOD1, and PRDX1 through a "peroxisome antioxidant-oxidant stress" signaling pathway to regulate the antioxidant and anti-stress ability of Tibetan pig liver.

### Peroxisome biosynthesis and mRNA expression of key antioxidant genes and abundance of key antioxidant enzymes

**Fig 6:**
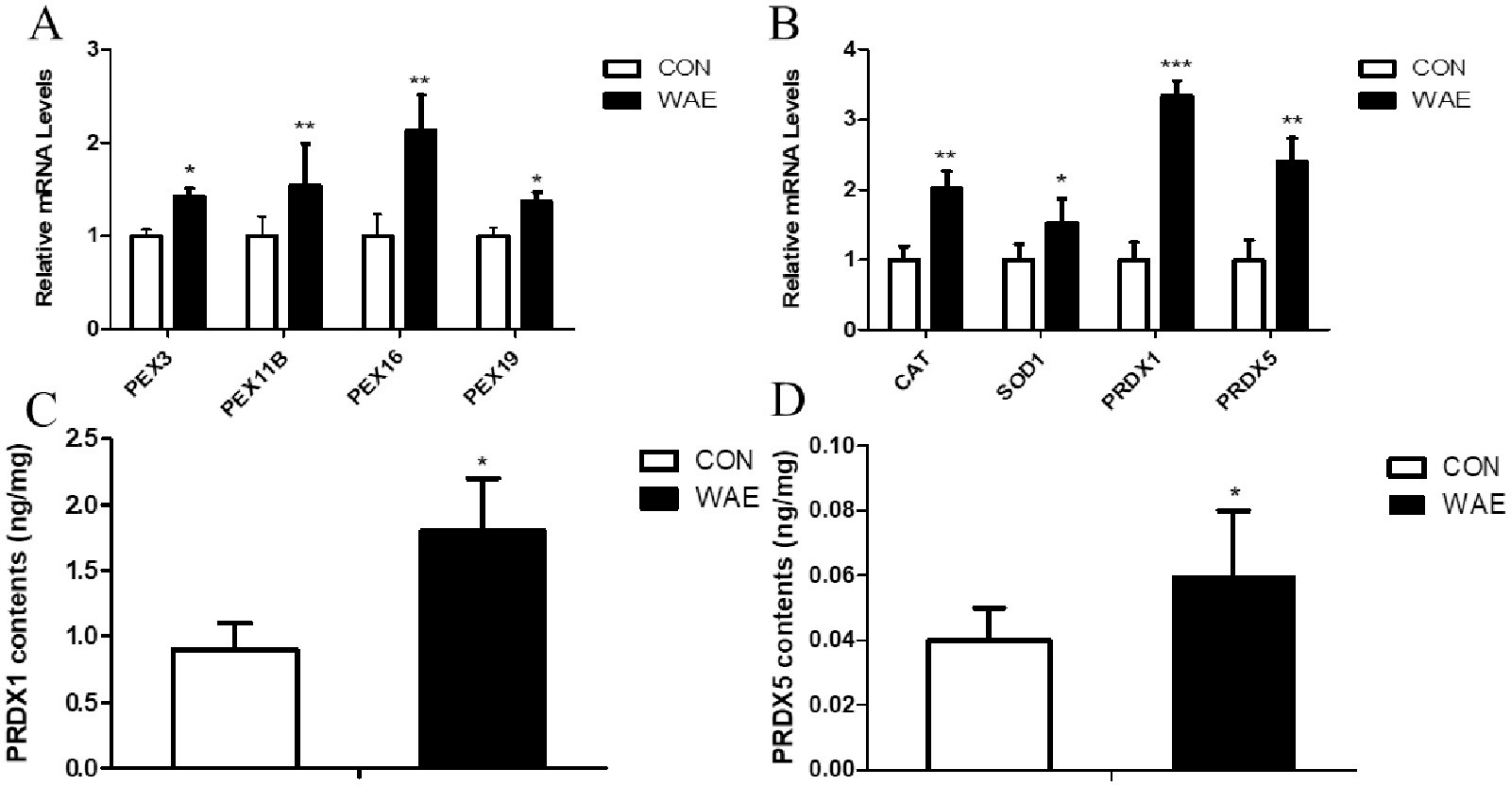
qPCR and ELISA were used to detect liver tissues PEX3, PEX11B, PEX16, PEX19, CAT, SOD1 and other key genes and PRDX1, PRDX5 protein content. (A) mRNA expression levels of key genes in peroxisome biosynthesis in the liver; (B) mRNA expression levels of key peroxisome antioxidant genes in the liver; (C) Peroxiredoxin 1 (PRDX1) content in the liver; (D) Peroxiredoxin 5 (PRDX5) content in the liver; *: (P<0.05) **: (P<0.01) ***: (P<0.001).

We analyzed the expression of key peroxisome biosynthesis genes PEX3, PEX11B, PEX16, and PEX19 and key peroxisome antioxidant genes CAT, SOD1, PRDX1, and PRDX5 in peroxisome biosynthesis by qPCR. The ELISA detected the presence of PRDX1 and PRDX5. The results showed that the four peroxisome biosynthesis genes in the WAE group were up-regulated; among them PEX11B and PEX19 were significantly differentially (P <0.05) (Fig 6A). The expression of the four antioxidant genes were all significantly up-regulated, (Fig 6B), and the content of PRDX1 and PRDX5 was increased significantly (Fig 6C and 6D).

## Discussion

Plant extracts are proposed to be used as antioxidants in animal feed, which will protect animals from oxidative damage caused by free radicals. The antioxidative properties of extracts of oregano, thyme, clove, pepper, lavender, and basil have been evaluated by many studies in vitro [33–36]. Slamenova et al. indicated that carvacrol given in drinking water reduced the level of DNA lesions induced in freshly isolated hepatocytes by H_2_O_2_, which could be associated with an increase of antioxidant activity of liver cells in these animals [37]. We found that WAE significantly increased the SOD1 and CAT activity and T-AOC, and reduce the MDA content. SOD is a scavenger oxygen free radical, CAT is a scavenger of hydrogen peroxide, T-AOC encompasses the various antioxidant substances in tissues and reflects the total antioxidant level of enzymes, and MDA is the product of peroxidation. These four indicators indicate the antioxidant capacity of liver tissue. An iron supplement test in the iron-deficiency anemia mouse model, showed that the APS-iron (III) complex can quickly supplement iron, increase SOD and CAT activity, and reduce the normal MDA level [38]. LBP has been shown to reduce necrotizing inflammation and oxidative stress caused by chemical toxins [39]. Nagwa M. Elsawi et al. found that Lycium Barbarum Extract is effective in reducing oxidative stress induced by a chemical toxin. Thus, goji has a great potential use as a food supplement in protection of the liver from injuries due to exposure to toxic chemicals or other related insults [40]. In this study, WAE had a significant effect on the antioxidants in Tibetan pig liver. It improved the liver antioxidant indicators, and t the anti-stress ability of the liver from the WAE group was significantly stronger than it was in the control group.

To determine the effect of WAE on the antioxidant genes in Tibetan pig liver, we used DESeq2 to identify the DEGs between the control and WAE groups. We found 3739 DEGS; 1843 were up-regulated and 1896 were down-regulated. According to the criteria of P <0.05 and |log2FC| >1, and the results of the functional annotation, we identified a total of 106 DEGs related to oxidative stress; 42 were up-regulated and 64 were down-regulated. The GO enrichment analysis showed that the 106 DEGs were enriched mainly in positive regulation of reactive oxygen metabolism, regulation of reactive oxygen biosynthesis, response to oxidation, regulation of stress response, cell response to oxidative stress, regulation of oxidoreductase activity, and process of peroxide metabolism. The KEGG Pathway enrichment analysis results showed that the DEGs were enriched mainly in PI3K-Akt, AMPK, Rap1, peroxisome, Wnt, and IL-17 signaling pathways. Adenylate activated protein kinase (AMPK) regulates the energy production of glucose and fatty acids during stress, inhibits the synthesis of cholesterol and glycogen, and the energy consumption of proteins [41, 42]. Salminen et al. found that the role of AMPK was not limited to the maintenance of energy metabolism when energy expenditure increased, rather AMPK also regulated oxidative stress, endoplasmic reticulum stress, autophagy, and inflammation to increase the anti-stress ability of cells [43]. AMPK also plays a key role in the complex signal network that regulates mitochondrial biogenesis. The function of mitochondria decreases with age and the rate of renewal slows down, leading to further accumulation of modified proteins, lipids, and DNA, aggravating mitochondrial insufficiency and leading to cell apoptosis [44]. The phosphatidylinositol 3’-kinase (PI3K-Akt) signaling pathway plays a key role in regulating the survival signals of various cells. The recent identification of multiple substrates of serine/threonine kinase Akt showed that it prevented cell death by targeting the cytoplasmic cell death mechanism and regulating the expression of cell death and survival-related genes [45]. However, the number of DEGs enriched in these two signaling pathways was relatively small, and they were mainly enriched in peroxisomes.

Peroxisomes are monolayer membrane organelles that are commonly found in various types of eukaryotic cells. They are very important multifunctional organelles involved in the dynamic rotation of ROS generation and removal, fatty acid oxidation, β-oxidation of long-chain fatty acids, decomposition of purines, and glycerol and bile acid biosynthesis [46]. Peroxisomes are independent organelles that are produced through "growth and division". They grow by introducing matrix and membrane proteins from the cytoplasm after translation, and then reproduce by dividing the original organelles; however, under special circumstances they can be formed from the endoplasmic reticulum from scratch [47–49]. Some metabolic processes of peroxisomes are completed in coordination with mitochondria, such as fatty acid β-oxidation and amino acid metabolism, but the oxidation of various substrates is completed by oxidases that consume oxygen. The peroxidase produced peroxidase Hydrogen is a by-product, which is broken down by the peroxisome labeling enzyme catalase and other antioxidant enzymes [50, 51]. Peroxisomes are known to play a key role in oxidative metabolism and oxidation balance because they have been shown to consume 20% of the total oxygen consumption and produce 35% of the cellular hydrogen peroxide [52].

**Fig 7:**
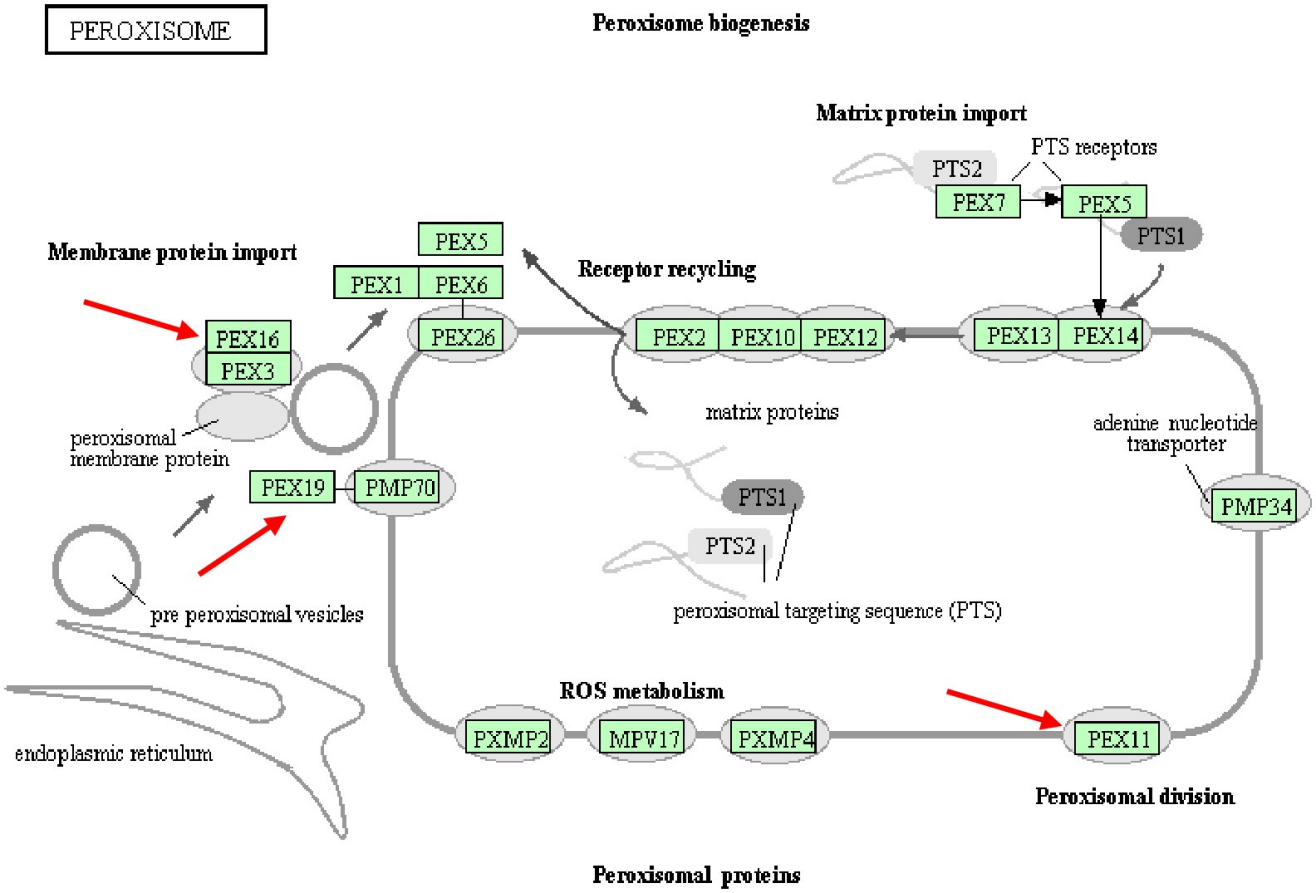
Peroxidase signaling pathway map. The gene indicated by the arrow is a key gene for peroxisome biosynthesis, and the WAE group and the control group are differentially expressed.

We used the KEGG Pathway database to obtain a peroxisome pathway map that contained information about 76 main genes (Fig 7). The mRNA expression levels of key genes PEX3, PEX11B, PEX16, and PEX19 involved in peroxisome biosynthesis were determined by RNA-seq analysis and qPCR. All four genes were up-regulated, and PEX11B and PEX16 were significantly up-regulated (P <0.01). Studies have found that oxidative stress may be related to the proliferation of peroxisomes based on "growth and division", whereby peroxisomes undergo a series of clear morphological changes, including growth, contraction, and final division. PEX11B is involved in the growth of peroxisomes (microtubules), and the growth of peroxisomes is caused by the production of ROS in animal cells [53]. Moreover, net formation of the peroxisomal membrane from a certain membrane was shown to be essential in the mutants defective in one of the three PEX genes (PEX3, PEX16, and PEX19) because these mutations lack peroxisomal ghosts but nevertheless are complemented for peroxisome biogenesis by the transfer of respective genes [54, 55]. Mammalian PEX3 acts directly on peroxisomes through PEX16/PEX19, and acts directly on the endoplasmic reticulum through PEX16-dependent and PEX19-independent pathways [56, 57]. Together, these results indicate that the PEX genes are key in the synthesis and assembly of peroxisomes, and their different expression levels between the WAE and control groups reflect the ability and quantity of liver peroxisome formation.

The main functions of peroxisomes are fatty acid oxidation, ether lipid synthesis, and antioxidant systems that include CAT, SOD, PRDX1, and PRDX5. Because peroxisomes can produce up to 35% of the cellular hydrogen peroxide, hydrogen peroxide is an important signal molecule that participates in the regulation of cell proliferation, apoptosis, and carbohydrate metabolism. However, at high concentrations hydrogen peroxide is toxic, so its concentration levels need to be strictly controlled. CAT is the most significant enzyme in peroxisomes and is involved mainly in this process [5, 58]. SOD1 is considered to be a true peroxisome protein, a powerful superoxide scavenger that catalyzes the conversion of oxygen to superoxide anion (O2^−^) [59]. Our results showed that the expression of CAT, SOD1, PRDX1, and PRDX5 in the liver tissue of the WAE group was significantly up-regulated and the PRDX1 and PRDX5 content was significantly higher than in the control group. Peroxidases (PRDXs) are important antioxidant proteins that form a powerful defense system that maintains redox balance by converting hydrogen peroxide to water [60]. PRDX1 belongs to the PRDX family and is a 23-kDa stress-induced macrophage redox protein that has multiple functions, including managing hydrogen peroxide-mediated oxidative stress in vitro and in vivo, and is a potential target for breast cancer treatment [61–63]. Recently, it was shown that PRDX1 can reduce ROS and inhibit apoptosis induced by the MAPK signaling pathway [64, 65]. PRDX5 also belongs to the PRDX family and is essential for regulating oxidative stress [66]. The cell protection function of PRDX5 has been fully proven. The structures and catalytic mechanisms of PRDX5 are different from those of the other PRDXs. For example, human PRDX5 has broader substrate specificity and can reduce alkyl hydroperoxides and peroxynitrite and hydrogen peroxide, has a wide range of sub-cell distribution, and is confined to mitochondria, cytoplasm, and peroxisomes, and, under certain circumstances, is located in the nucleus [67]. Adenoviral vectors have been used to overexpress PRDX5 in liver transplants to assess its protective effect. The results showed that PRDX5 overexpression reduced the damage to small liver transplants and improved the survival rate of recipients [68].

**Fig 8:**
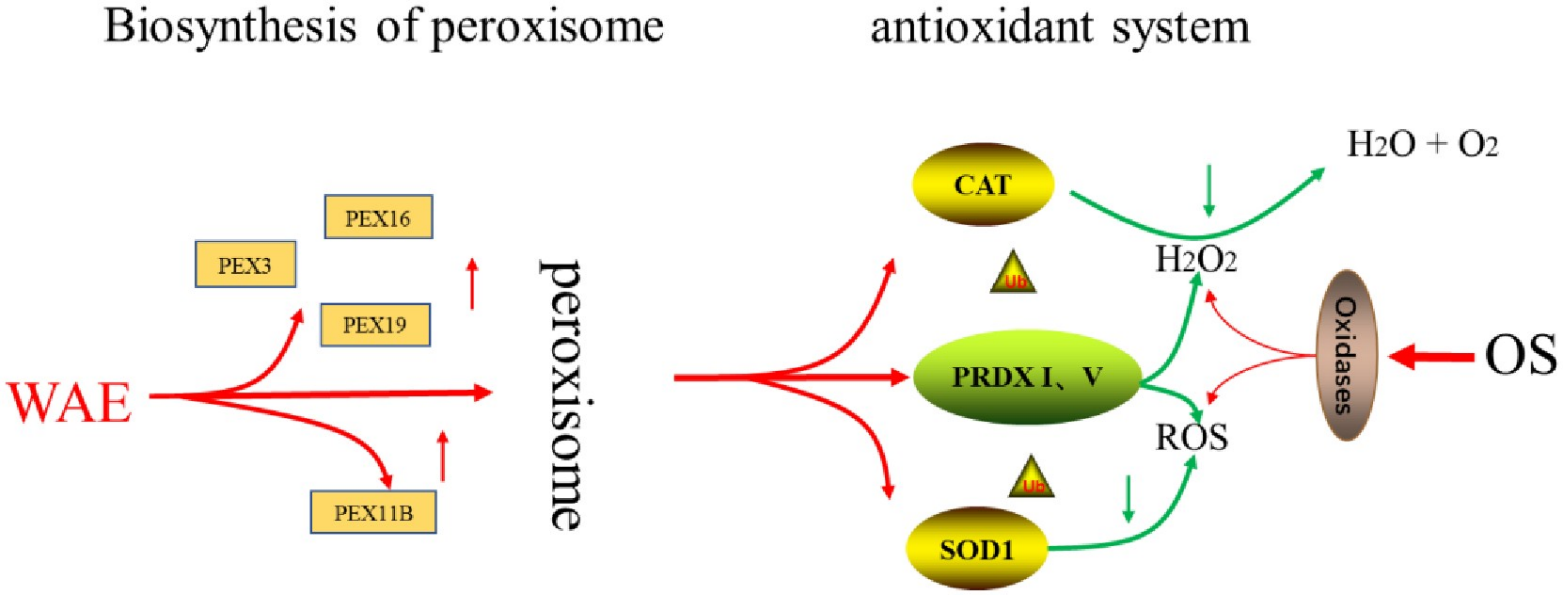
Regulation of WEA on antioxidant mechanism of Tibetan pig liver.

The four key antioxidant proteases, CAT, SOD1, PRDX1, and PRDX5, constitute the antioxidant system of the peroxisome signaling pathway because of their unique roles as antioxidants. Our results showed that the mRNA expression levels of these four key genes were up-regulated in the WAE group compared with in the control group. The CAT and SOD1 activity were significantly higher than in the control group, and the abundance of PRDX1 and PRDX5 was significantly higher than in the control group. The mRNA expression levels of the key genes in peroxidase biosynthesis indicated that the number and ability of peroxisome production were significantly stronger in the WAE group than in the control group. This led to changes in the peroxisome marker antioxidant CAT and SOD1 activity and changes in the content of key antioxidant proteins PRDX1 and PRDX5, thereby improving the antioxidation and anti-stress ability of Tibetan pig liver, as shown in Fig 8.

## Conclusions

The WAE regulated the antioxidant and anti-stress ability of Tibetan pig liver through a “peroxisome antioxidant-oxidant stress" signaling pathway. We propose that WAE can be used as a healthy and green antioxidant to protect the liver of Tibetan pigs.

## Supporting information

S1 Table Basic diet composition (%) and nutrient level (g/100g). (DOCX)

S2 Table Primer sequences of the target and reference genes. (DOCX)

S3 Table EST analysis of differential gene expression. (DOCX)

## Abbreviations

AMPK: Adenylate activated protein kinase
CAT: catalase
DEGs: differentially expressed genes
ELLSA: Enzyme Linked Immunosorbent Assay
HEP90AA1: heat shock protein 90 family Amember1
LBP: lycium barbarum polysaccharides
MDA: malondialdehyde
PARK7: Parkinson’s syndrome related deglycase 7
PEX11B: peroxisomal biogenesis factor 11B
PEX16: peroxisomal biogenesis factor 16
PEX19: peroxisomal biogenesis factor19
PEX3: peroxisomal biogenesis factor 3
PI3K-Akt: phosphatidylinositol 3’-kinase
PRDX1: Peroxidase1
PRDX5: peroxidase5
RIN: RNA integrity number
RNA-seq: transcriptome sequencing
ROS: reactive oxygen species
SOD1: superoxide dismutase1
T-SOD: total antioxidative capacity
WAE: wolfberry and Astragalus extract
