# Supplementary material for: Effects of Chinese wolfberry and Astragalus extract on the antioxidant capacity of Tibetan pig liver
Source: PLoS One. 2021 Jan 27;16(1):e0245749. doi: 10.1371/journal.pone.0245749 (PMC7840052; doi:10.1371/journal.pone.0245749)
Supplement: S1 Table — (DOCX) [file pone.0245749.s001.docx]

**S1 Table．** Basic diet composition (%) and nutrient level (g/100g)

| **Items** | **Content** |
| --- | --- |
| Ingredients |  |
| Corn  Soybean meal | 35.00  46.00 |
| Wheat flour | 11.50 |
| Rice bran | 3.95 |
| Soybean oil | 1.20 |
| Ca_3_(PO_4_)_2_ | 0.45 |
| Limestone | 0.90 |
| Premix^1^  Total | 1.00  100.00 |
| Nutrient levels  DM | 87.24 |
| CP  CF  NDF  ADF | 13.78  4.80  15.32  4.68 |
| Ca | 0.99 |
| TP | 0.56 |
| Lys | 0.91 |
| Met  DE^2^(MJ/kg) | 0.25  13.20 |

DM: dry matter CP: crude protein; CF: crude fat; NDF: neutral detergent fibre; and ADF: acid detergent fibre.

^1^ Premix： 12 500 IU vitamin A; 4 500 IU vitamin D_3_; 25 IU vitamin E; 3 mg vitamin B_1_ mg; 7 mg vitamin B_2_; 10 mg vitamin B_5_; 7 mg vitamin B_6_; 0.2 mg vitamin B_12;_ 0.15 mg biotin; 1.1 mg folic acid; 10 mg Cu; 50 mg Fe; 100 mg Mn; 85 mg Zn; 0.3 mg Se.

^2^ DE: Nutrient levels were calculated values.
