# Supplementary material for: Effects of Chinese wolfberry and Astragalus extract on the antioxidant capacity of Tibetan pig liver
Source: PLoS One. 2021 Jan 27;16(1):e0245749. doi: 10.1371/journal.pone.0245749 (PMC7840052; doi:10.1371/journal.pone.0245749)
Supplement: S2 Table — (DOCX) [file pone.0245749.s002.docx]

**S2 Table．**Primer sequences of the target and reference genes.

| **gene** | **Accession number** | **Primers sequence** | | **Product Size/bp** | |
| --- | --- | --- | --- | --- | --- |
| ACTB | [XM_021086047.1](https://www.ncbi.nlm.nih.gov/nuccore/XM_021086047.1) | FORWARD | 5’-CTACGCCAACACGGTGCTGTC-3’ | 325 |  |
|  |  | REVERSE | 5’-CTCCTGCTTGCTGATCCACATCTG-3’ |  |  |
| PEX3 | [NM_001244185.1](https://www.ncbi.nlm.nih.gov/nuccore/NM_001244185.1) | FORWARD | 5’-GCCCAAGCACGACGACAGTATC-3’ | 88 |  |
|  |  | REVERSE | 5’-CCTCTCTCAGTGTTGGAAGCATGG-3’ |  |  |
| PEX11B | [XM_001927969.4](https://www.ncbi.nlm.nih.gov/nuccore/XM_001927969.4) | FORWARD | 5’-GGCTCTGAAGTTTCGGCTCCAAG-3’ | 93 |  |
|  |  | REVERSE | 5’-GGCTCTGAAGTTTCGGCTCCAAG-3’ |  |  |
| PEX16 | [XM_003122832.4](https://www.ncbi.nlm.nih.gov/nuccore/XM_003122832.4) | FORWARD | 5’-AGGTGTGATGGACAGGGTGGTG-3’ | 99 |  |
|  |  | REVERSE | 5’-TCTGGTCTATTGGTGGCTCCGATC-3’ |  |  |
| PEX19 | [XM_001928869.5](https://www.ncbi.nlm.nih.gov/nuccore/XM_001928869.5) | FORWARD | 5’-AAGAATGCCACCGACCTTCAGAAC-3‘ | 80 |  |
|  |  | REVERSE | 5’-TCTTCCATGCCCAGCCCTTCC-3’ |  |  |
| PRDX1 | [XM_021096742.1](https://www.ncbi.nlm.nih.gov/nuccore/XM_021096742.1) | FORWARD | 5’-CCGCTCCGTGGATGAGACTCTG-3’ | 99 |  |
|  |  | REVERSE | 5’-GTATCACTGCCAGGCTTCCAACC-3’ |  |  |
| PRDX5 | [NM_214144.1](https://www.ncbi.nlm.nih.gov/nuccore/NM_214144.1) | FORWARD | 5’-CATCGGTGGTGGTGTTTGAAGGG-3’ | 124 |  |
|  |  | REVERSE | 5’-TGGGTCTTGGAACAGCCAGGAG-3’ |  |  |
| CAT | [NM_214301.2](https://www.ncbi.nlm.nih.gov/nuccore/NM_214301.2) | FORWARD | 5’-GCCGCCTATTTGCCTATCCTGAC-3’ | 85 |  |
|  |  | REVERSE | 5’-GCACGGAAGGGACAGTTCACAG-3’ |  |  |
| SOD1 | [NM_001190422.1](https://www.ncbi.nlm.nih.gov/nuccore/NM_001190422.1) | FORWARD | 5’-GAAGATTCTGTGATCGCCCTCTCG-3’ | 99 |  |
|  |  | REVERSE | 5’-TTCATTTCCACCTCTGCCCAAGTC-3’ |  |  |
| HSP90AA1 | [NM_213973.2](https://www.ncbi.nlm.nih.gov/nuccore/NM_213973.2) | FORWARD | 5’TCACCGTTAGGACAGACACAGGAG-3’ | 182 |  |
|  |  | REVERSE | 5’-TCTTCCGCCTCGTCGTCACTG-3’ |  |  |
| HAO1 | [XM_021077275.1](https://www.ncbi.nlm.nih.gov/nuccore/XM_021077275.1) | FORWARD | 5’-CCTTACCTGGGCAACCGCTTTG-3’ | 234 |  |
|  |  | REVERSE | 5’-ACCTCCTTGGCATCATCACCTCTC-3’ |  |  |
| HADH | [NM_214331.1](https://www.ncbi.nlm.nih.gov/nuccore/NM_214331.1) | FORWARD | 5’-GCCAATGCCACCACCAGACAG-3’ | 229 |  |
|  |  | REVERSE | 5’-GATGCGTCACCTCGTTCGTACAG-3’ |  |  |
| DECR1 | [NM_001190232.2](https://www.ncbi.nlm.nih.gov/nuccore/NM_001190232.2) | FORWARD | 5’-TAGCCGTCTTGACCCAACTGGAG-3’ | 190 |  |
|  |  | REVERSE | 5’-CCTTGGTAACCTTCCGCAGATGG-3’ |  |  |
| ACADVL | [XM_021067809.1](https://www.ncbi.nlm.nih.gov/nuccore/XM_021067809.1) | FORWARD | 5’-GCCCGCTTGGTGGAGATTGTC-3’ | 309 |  |
|  |  | REVERSE | 5’-GCTGGGTCTGTAACTGGTGTCTTG-3’ |  |  |
| SESN2 | [XM_021095666.1](https://www.ncbi.nlm.nih.gov/nuccore/XM_021095666.1) | FORWARD | 5’-TTGTCTTTGGCTGTGGCATCCTC-3’ | 210 |  |
|  |  | REVERSE | 5’-GCGGCTCTCCATCTCCTCCTG-3’ |  |  |
| MSRB1 | [NM_001097460.1](https://www.ncbi.nlm.nih.gov/nuccore/NM_001097460.1) | FORWARD | 5’-CCAGAACCACTTTGAGCCAGGTG-3’ | 83 |  |
|  |  | REVERSE | 5’-GCCCAGTCCGTTGCCACATC-3’ |  |  |
| PARK7 | [NM_001078663.1](https://www.ncbi.nlm.nih.gov/nuccore/NM_001078663.1) | FORWARD | 5’-TGCAGTGTAGCCGTGATGTTGTC-3’ | 232 |  |
|  |  | REVERSE | 5’-AGTGGGTGCGTCGTAACTTTGC-3’ |  |  |
