# Supplementary material for: Effects of Chinese wolfberry and Astragalus extract on the antioxidant capacity of Tibetan pig liver
Source: PLoS One. 2021 Jan 27;16(1):e0245749. doi: 10.1371/journal.pone.0245749 (PMC7840052; doi:10.1371/journal.pone.0245749)
Supplement: S3 Table — (DOCX) [file pone.0245749.s003.docx]

**S3 Table**. EST analysis of differential gene expression

| **Gene bank Accession** | **Symbol** | **log2FoldChange** | **p-value** |
| --- | --- | --- | --- |
| [NP_008646.1](https://www.ncbi.nlm.nih.gov/protein/NP_008646.1) | CYTB | 2.061764 | 9.55E-72 |
| [NP_008642.1](https://www.ncbi.nlm.nih.gov/protein/NP_008642.1) | ND4L | 2.11983 | 1.65E-70 |
| [NP_008635.1](https://www.ncbi.nlm.nih.gov/protein/NP_008635.1) | ND2 | 1.925287 | 2.06E-56 |
| [NP_008634.1](https://www.ncbi.nlm.nih.gov/protein/NP_008634.1) | ND1 | 1.976677 | 2.02E-52 |
| [NP_008641.1](https://www.ncbi.nlm.nih.gov/protein/NP_008641.1) | ND3 | 1.849999 | 1.42E-47 |
| [NP_008640.1](https://www.ncbi.nlm.nih.gov/protein/NP_008640.1) | COX3 | 1.73268 | 6.10E-42 |
| [NP_008636.1](https://www.ncbi.nlm.nih.gov/protein/NP_008636.1) | COX1 | 1.914683 | 8.33E-36 |
| [NP_008644.1](https://www.ncbi.nlm.nih.gov/protein/NP_008644.1) | ND5 | 2.053706 | 1.46E-35 |
| [NM_214064.1](https://www.ncbi.nlm.nih.gov/nuccore/NM_214064.1) | FMO1 | -1.90851 | 4.46E-35 |
| [NP_008637.1](https://www.ncbi.nlm.nih.gov/protein/NP_008637.1) | COX2 | 1.60442 | 5.46E-33 |
| [NP_008643.1](https://www.ncbi.nlm.nih.gov/protein/NP_008643.1) | ND4 | 1.866313 | 1.53E-31 |
| [NM_001044611.2](https://www.ncbi.nlm.nih.gov/nuccore/NM_001044611.2) | ALDH2 | -1.26569 | 1.82E-31 |
| [XM_001925224.6](https://www.ncbi.nlm.nih.gov/nuccore/XM_001925224.6) | TP53INP1 | -1.82387 | 2.92E-30 |
| [XM_003130094.5](https://www.ncbi.nlm.nih.gov/nuccore/XM_003130094.5) | FMO3 | -4.81822 | 1.39E-27 |
| [NP_008645.1](https://www.ncbi.nlm.nih.gov/protein/NP_008645.1) | ND6 | 2.128027 | 1.61E-27 |
| [NM_214050.2](https://www.ncbi.nlm.nih.gov/nuccore/NM_214050.2) | GSTO1 | -1.17782 | 7.48E-25 |
| [NM_001244900.1](https://www.ncbi.nlm.nih.gov/nuccore/NM_001244900.1) | AASS | -1.25196 | 4.45E-19 |
| [NM_214426.1](https://www.ncbi.nlm.nih.gov/nuccore/NM_214426.1) | CYP8B1 | -3.95273 | 1.63E-18 |
| [NM_214412.1](https://www.ncbi.nlm.nih.gov/nuccore/NM_214412.1) | CYP1A1 | -1.92946 | 5.91E-17 |
| [XM_005655126.3](https://www.ncbi.nlm.nih.gov/nuccore/XM_005655126.3) | DECR2 | 0.937582 | 2.10E-16 |
| [XM_005663076.3](https://www.ncbi.nlm.nih.gov/nuccore/XM_005663076.3) | CYP7A1 | -2.2692 | 5.97E-16 |
| [NM_001110426.1](https://www.ncbi.nlm.nih.gov/nuccore/NM_001110426.1) | PEX6 | -1.02358 | 3.71E-15 |
| [NM_001113041.1](https://www.ncbi.nlm.nih.gov/nuccore/NM_001113041.1) | FADS1 | 1.768103 | 1.39E-13 |
| [XM_003133603.5](https://www.ncbi.nlm.nih.gov/nuccore/XM_003133603.5) | NDUFS1 | -0.87076 | 7.47E-13 |
| [XM_001927795.7](https://www.ncbi.nlm.nih.gov/nuccore/XM_001927795.7) | HSPA5 | -2.71471 | 5.43E-12 |
| [XM_003123324.4](https://www.ncbi.nlm.nih.gov/nuccore/XM_003123324.4) | ASNA1 | -0.9351 | 1.50E-11 |
| [NM_001244474.1](https://www.ncbi.nlm.nih.gov/nuccore/NM_001244474.1) | PRDX2 | -0.91569 | 1.69E-11 |
| [NM_214416.1](https://www.ncbi.nlm.nih.gov/nuccore/NM_214416.1) | IYD | -0.81608 | 2.13E-11 |
| NM_213973.2 | HSP90AA1 | -0.85005 | 9.36E-11 |
| [XM_021079475.1](https://www.ncbi.nlm.nih.gov/nuccore/XM_021079475.1) | AIFM1 | -0.84619 | 1.33E-10 |
| [NM_001244162.1](https://www.ncbi.nlm.nih.gov/nuccore/NM_001244162.1) | SORD | -0.85204 | 1.63E-10 |
| [NM_001078663.1](https://www.ncbi.nlm.nih.gov/nuccore/NM_001078663.1) | PARK7 | -0.6789 | 1.81E-09 |
| [NM_001244398.1](https://www.ncbi.nlm.nih.gov/nuccore/NM_001244398.1) | PDHB | -0.77368 | 5.11E-09 |
| [XM_003126560.5](https://www.ncbi.nlm.nih.gov/nuccore/XM_003126560.5) | NDUFA9 | -0.80198 | 8.36E-09 |
| [NM_001144842.2](https://www.ncbi.nlm.nih.gov/nuccore/NM_001144842.2) | NDUFB8 | -0.96016 | 2.59E-08 |
| [XM_013989414.2](https://www.ncbi.nlm.nih.gov/nuccore/XM_013989414.2) | HELQ | 1.259184 | 7.47E-08 |
| [NM_001044556.1](https://www.ncbi.nlm.nih.gov/nuccore/NM_001044556.1) | MGST3 | -0.92718 | 2.50E-07 |
| [NM_001001640.1](https://www.ncbi.nlm.nih.gov/nuccore/NM_001001640.1) | MAOA | -0.96435 | 3.16E-07 |
| [NM_000492.4](https://www.ncbi.nlm.nih.gov/nuccore/NM_000492.4) | CFTR | -2.77375 | 4.68E-07 |
| [NM_001195119.1](https://www.ncbi.nlm.nih.gov/nuccore/NM_001195119.1) | PDIA6 | -1.00816 | 5.37E-07 |
| [XM_003126364.4](https://www.ncbi.nlm.nih.gov/nuccore/XM_003126364.4) | HELB | -1.11076 | 6.17E-07 |
| [XM_013995763.2](https://www.ncbi.nlm.nih.gov/nuccore/XM_013995763.2) | CIAO3 | 0.636663 | 8.60E-07 |
| [NM_001097460.1](https://www.ncbi.nlm.nih.gov/nuccore/NM_001097460.1) | MSRB1 | 0.747218 | 9.06E-07 |
| [NM_214201.1](https://www.ncbi.nlm.nih.gov/nuccore/NM_214201.1) | GPX1 | -0.91943 | 9.09E-07 |
| [XM_003361882.4](https://www.ncbi.nlm.nih.gov/nuccore/XM_003361882.4) | NDUFB4 | -0.65194 | 1.39E-06 |
| [XM_003133964.6](https://www.ncbi.nlm.nih.gov/nuccore/XM_003133964.6) | MTREX | -0.76523 | 1.50E-06 |
| [NM_001005157.1](https://www.ncbi.nlm.nih.gov/nuccore/NM_001005157.1) | DDX39B | 0.763719 | 1.56E-06 |
| [XM_003356899.5](https://www.ncbi.nlm.nih.gov/nuccore/XM_003356899.5) | UGDH | 0.716409 | 1.85E-06 |
| [XM_013988107.2](https://www.ncbi.nlm.nih.gov/nuccore/XM_013988107.2) | HAO2 | 0.584616 | 3.67E-06 |
| [XM_021073627.1](https://www.ncbi.nlm.nih.gov/nuccore/XM_021073627.1) | CTBP2 | 1.096836 | 5.81E-06 |
| [XM_003135126.3](https://www.ncbi.nlm.nih.gov/nuccore/XM_003135126.3) | HSD17B10 | -0.69622 | 5.93E-06 |
| [XM_001927529.6](https://www.ncbi.nlm.nih.gov/nuccore/XM_001927529.6) | ALDH18A1 | 1.063592 | 6.02E-06 |
| [NM_214249.1](https://www.ncbi.nlm.nih.gov/nuccore/NM_214249.1) | ATP1A1 | -0.67436 | 9.27E-06 |
| [XM_005671928.3](https://www.ncbi.nlm.nih.gov/nuccore/XM_005671928.3) | CYBRD1 | -0.92384 | 9.43E-06 |
| [XM_003358607.4](https://www.ncbi.nlm.nih.gov/nuccore/XM_003358607.4) | PFN2 | -1.01967 | 9.67E-06 |
| [XM_013991587.2](https://www.ncbi.nlm.nih.gov/nuccore/XM_013991587.2) | CP | -0.58919 | 1.60E-05 |
| [NM_214411.1](https://www.ncbi.nlm.nih.gov/nuccore/NM_214411.1) | COX7A1 | -0.73712 | 3.74E-05 |
| [XM_003355917.4](https://www.ncbi.nlm.nih.gov/nuccore/XM_003355917.4) | BLVRB | 0.514261 | 4.42E-05 |
| [XM_003123645.5](https://www.ncbi.nlm.nih.gov/nuccore/XM_003123645.5) | LTC4S | 0.631774 | 8.50E-05 |
| [XM_021067809.1](https://www.ncbi.nlm.nih.gov/nuccore/XM_021067809.1) | ACADVL | 0.418792 | 9.46E-05 |
| [NM_001190232.2](https://www.ncbi.nlm.nih.gov/nuccore/NM_001190232.2) | DECR1 | 0.495713 | 0.000123 |
| [NM_213865.1](https://www.ncbi.nlm.nih.gov/nuccore/NM_213865.1) | ATP2A2 | -0.77554 | 0.000145 |
| [NM_001244428.1](https://www.ncbi.nlm.nih.gov/nuccore/NM_001244428.1) | CLIC2 | -1.10451 | 0.000163 |
| [XM_003129944.4](https://www.ncbi.nlm.nih.gov/nuccore/XM_003129944.4) | SC5D | -0.5893 | 0.000166 |
| [XM_021077477.1](https://www.ncbi.nlm.nih.gov/nuccore/XM_021077477.1) | TRMT9B | 1.107522 | 0.000194 |
| [XM_001928249.4](https://www.ncbi.nlm.nih.gov/nuccore/XM_001928249.4) | TMX1 | -1.41543 | 0.000212 |
| [NM_001243431.1](https://www.ncbi.nlm.nih.gov/nuccore/NM_001243431.1) | HAO1 | -0.50714 | 0.00024 |
| [XM_005656949.3](https://www.ncbi.nlm.nih.gov/nuccore/XM_005656949.3) | VAT1 | -0.44314 | 0.000263 |
| [NM_002133.3](https://www.ncbi.nlm.nih.gov/nuccore/NM_002133.3) | HMOX1 | -0.53263 | 0.000282 |
| [NM_001167635.1](https://www.ncbi.nlm.nih.gov/nuccore/NM_001167635.1) | IVD | -0.50112 | 0.000283 |
| [NM_214166.1](https://www.ncbi.nlm.nih.gov/nuccore/NM_214166.1) | DHDH | 1.96331 | 0.000373 |
| [NM_001172363.2](https://www.ncbi.nlm.nih.gov/nuccore/NM_001172363.2) | LDHA | -0.44457 | 0.000486 |
| [NM_001129959.1](https://www.ncbi.nlm.nih.gov/nuccore/NM_001129959.1) | POR | 0.586291 | 0.000499 |
| [XM_003134698.3](https://www.ncbi.nlm.nih.gov/nuccore/XM_003134698.3) | ATP6V1F | -0.55785 | 0.000651 |
| [NM_001243354.1](https://www.ncbi.nlm.nih.gov/nuccore/NM_001243354.1) | DHCR24 | -0.46333 | 0.00075 |
| [NM_001206403.1](https://www.ncbi.nlm.nih.gov/nuccore/NM_001206403.1) | LOX | -1.05671 | 0.000847 |
| [NM_001123106.1](https://www.ncbi.nlm.nih.gov/nuccore/NM_001123106.1) | DHX16 | 0.491519 | 0.000904 |
| [NM_001164113.1](https://www.ncbi.nlm.nih.gov/nuccore/NM_001164113.1) | SELENOS | -0.69045 | 0.000908 |
| [XM_005655085.3](https://www.ncbi.nlm.nih.gov/nuccore/XM_005655085.3) | HSD3B7 | -0.53657 | 0.001005 |
| [NM_001131046.1](https://www.ncbi.nlm.nih.gov/nuccore/NM_001131046.1) | IFI30 | -0.84654 | 0.001103 |
| [XM_003480775.4](https://www.ncbi.nlm.nih.gov/nuccore/XM_003480775.4) | NDUFB7 | 0.425776 | 0.001224 |
| [XM_003124557.5](https://www.ncbi.nlm.nih.gov/nuccore/XM_003124557.5) | CRYM | -0.96339 | 0.001424 |
| [XM_021065541.1](https://www.ncbi.nlm.nih.gov/nuccore/XM_021065541.1) | KATNAL1 | 0.772907 | 0.001526 |
| [XM_005655176.3](https://www.ncbi.nlm.nih.gov/nuccore/XM_005655176.3) | ABCA3 | 0.578274 | 0.001645 |
| [XM_003135200.4](https://www.ncbi.nlm.nih.gov/nuccore/XM_003135200.4) | ATP7A | -0.98758 | 0.001978 |
| [NM_214267.1](https://www.ncbi.nlm.nih.gov/nuccore/NM_214267.1) | CYBA | -0.57309 | 0.002343 |
| [NM_214343.2](https://www.ncbi.nlm.nih.gov/nuccore/NM_214343.2) | QDPR | -0.43564 | 0.002508 |
| [NM_213931.1](https://www.ncbi.nlm.nih.gov/nuccore/NM_213931.1) | ALOX15 | 1.220657 | 0.002537 |
| [NM_213874.1](https://www.ncbi.nlm.nih.gov/nuccore/NM_213874.1) | MDH1 | -0.41361 | 0.002723 |
| [NM_001315792.1](https://www.ncbi.nlm.nih.gov/nuccore/NM_001315792.1) | CYP26A1 | 1.36775 | 0.003285 |
| [XM_003121588.5](https://www.ncbi.nlm.nih.gov/nuccore/XM_003121588.5) | INO80 | -0.53145 | 0.003333 |
| [XM_003353343.5](https://www.ncbi.nlm.nih.gov/nuccore/XM_003353343.5) | MYO1E | -0.86574 | 0.003408 |
| [NM_001097522.1](https://www.ncbi.nlm.nih.gov/nuccore/NM_001097522.1) | CYB561 | -1.48827 | 0.004318 |
| [XM_003127577.5](https://www.ncbi.nlm.nih.gov/nuccore/XM_003127577.5) | PLOD1 | -0.48159 | 0.005358 |
| [XM_001929106.5](https://www.ncbi.nlm.nih.gov/nuccore/XM_001929106.5) | COQ6 | 0.490638 | 0.005833 |
| [XM_003127564.4](https://www.ncbi.nlm.nih.gov/nuccore/XM_003127564.4) | UBIAD1 | -0.74752 | 0.005923 |
| [XM_021095666.1](https://www.ncbi.nlm.nih.gov/nuccore/XM_021095666.1) | SESN2 | 0.508548 | 0.007188 |
| [XM_005667490.3](https://www.ncbi.nlm.nih.gov/nuccore/XM_005667490.3) | FOXRED1 | 0.312732 | 0.007785 |
| [XM_005670675.3](https://www.ncbi.nlm.nih.gov/nuccore/XM_005670675.3) | RFC5 | 0.551717 | 0.007828 |
| [NM_214331.1](https://www.ncbi.nlm.nih.gov/nuccore/NM_214331.1) | HADH | -0.33369 | 0.008004 |
| [NM_001044581.1](https://www.ncbi.nlm.nih.gov/nuccore/NM_001044581.1) | TAP1 | -0.47171 | 0.008485 |
| [NM_213915.2](https://www.ncbi.nlm.nih.gov/nuccore/NM_213915.2) | ATP5ME | 0.309726 | 0.008535 |
| [XM_003121325.4](https://www.ncbi.nlm.nih.gov/nuccore/XM_003121325.4) | RTN4IP1 | 0.372603 | 0.010564 |
| [NM_001244743.1](https://www.ncbi.nlm.nih.gov/nuccore/NM_001244743.1) | NDUFC2 | 0.417453 | 0.010939 |
| [XM_003355776.4](https://www.ncbi.nlm.nih.gov/nuccore/XM_003355776.4) | DDX28 | -0.83352 | 0.011053 |
| [XM_003480995.4](https://www.ncbi.nlm.nih.gov/nuccore/XM_003480995.4) | ALKBH4 | 0.488774 | 0.011927 |
